# Prognostic value of early intracranial pressure curve following surgical decompression: A machine learning analysis

**DOI:** 10.1016/j.bas.2026.106076

**Published:** 2026-05-02

**Authors:** Alim Emre Basaran, Cemalettin Göksu, Volker Thieme, Sebastian Stehr, Alexandru Guranda, Johannes Wach, Tim Wende, Erdem Güresir, Martin Vychopen

**Affiliations:** a Department of Neurosurgery, University Hospital Leipzig, 04103, Leipzig, Germany; b Department of Anesthesiology and Intensive Care, University Hospital Leipzig, Leipzig, Germany

**Keywords:** Intracranial pressure, Decompressive craniectomy, Neurological outcome prediction, Machine learning, Modified rankin scale

## Abstract

**Background:**

Intracranial pressure (ICP) control is a critical determinant of successful therapy following decompressive craniectomy (DC). While traditional assessments have focused on peak or mean ICP values, the prognostic relevance of continuously quantified ICP burden using the area under the curve (ICP-AUC) remains underexplored. This study aimed to evaluate the prognostic value of early postoperative ICP dynamics within the first 72 h using ICP-AUC based analysis and to assess predictive performance through machine learning models.

**Methods:**

Seventeen patients who underwent DC were included in this retrospective analysis. ICP was continuously recorded over a 72-h period after DC was performed. We calculated normalized ICP-AUC, maximum ICP values, and the ICP-AUC above thresholds was calculated separately based on baseline ICP values of 15 mmHg and 20 mmHg, respectively. Neurological outcomes were assessed using the modified Rankin Scale (mRS) at discharge. A poor outcome was defined as mRS = 6 (death).

**Results:**

A normalized ICP-AUC ≥0.5 was significantly associated with poor outcomes at discharge (Spearman r = 0.50, *p* = 0.04), and all patients with ICP-AUC ≥0.6 deceased. Furthermore, ICP levels exceeding 20 mmHg for more than 30% of the time were significantly associated with mRS = 6 (*p* = 0.02). Additional threshold analyses confirmed significance of ICP-AUC based on threshold of >15 mmHg (*p* = 0.047) >20 mmHg (*p* = 0.03). ROC analysis revealed an AUC of 0.89 for the 20 mmHg threshold, indicating high predictive accuracy. The Random Forest model achieved an AUC of 0.89, with a precision of 1.00 and an F1-score of 0.80. In contrast, the XGBoost model showed lower predictive values across all metrics (0.67). On the other hand, models based on a 15 mmHg ICP baseline demonstrated limited prognostic validity.

**Conclusion:**

Early cumulative ICP burden, assessed through ICP-AUC, might provide a reliable prognostic marker for neurological outcomes following DC. Machine learning enhances predictive accuracy and offers a promising approach for clinical decision support in neurocritical care.

## Highlights

• Early postoperative ICP-AUC within 72 h after decompressive craniectomy (DC) is a strong predictor of neurological outcome, outperforming traditional single ICP metrics such as mean or peak ICP.
• A normalized ICP-AUC ≥0.5 was significantly associated with mortality (mRS = 6), and all patients with ICP-AUC ≥0.6 died, identifying a clinically relevant threshold for risk stratification.
• ICP exceeding 20 mmHg for >30% of the monitoring period strongly predicted poor outcome, with significant associations across multiple statistical tests (*p* = 0.03).
• ROC analysis demonstrated high predictive accuracy (AUC = 0.89) for the 20 mmHg threshold, supporting its relevance as a prognostic marker in neurocritical care.
• Machine learning improved prognostic performance, with the Random Forest classifier achieving excellent precision (1.00) and high predictive accuracy (AUC = 0.89), outperforming XGBoost.
• ICP-AUC provides a dynamic, cumulative measure of ICP burden, capturing both magnitude and duration of intracranial hypertensionmoffering utility beyond traditional ICP monitoring approaches.

## Graphical abstract

**Figure:**
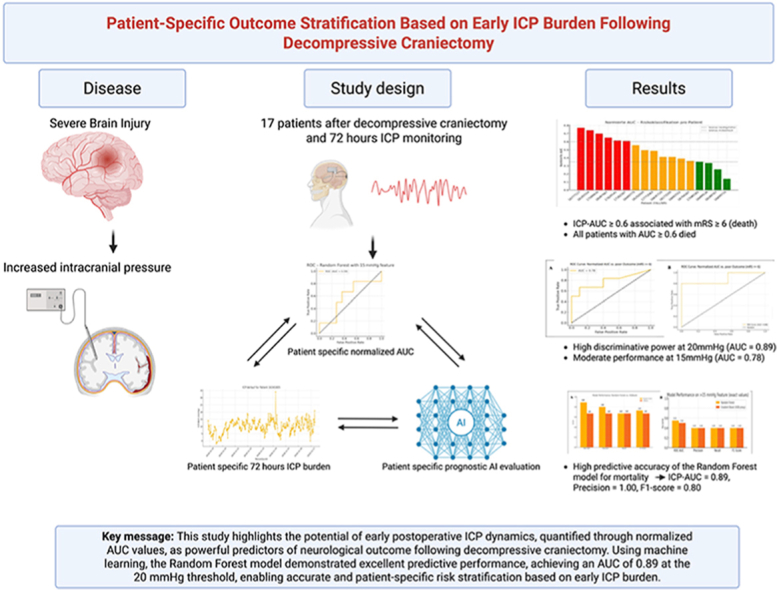

## 1 Introduction

Intracranial pressure (ICP) monitoring is a crucial component in the management of patients at risk of intracranial hypertension due to various pathologies such as malignant middle cerebral artery (MCA) infarction, subarachnoid hemorrhage (SAH), traumatic brain injury (TBI) and intracerebral hemorrhage (ICH) (Poca et al., 2010; Shim et al., 2023; Sauvigny et al., 2018; Chen et al., 2023). Particularly in patients undergoing decompressive craniectomy (DC), the prognostic value of postoperative ICP patterns after DC remains uncertain. While ICP decreases significantly after DC, the impact on the postoperative ICP burden remains unclear (Sauvigny et al., 2018). Postoperative ICP monitoring helps to prevent secondary brain damage by enabling timely neurosurgical interventions or targeted neurointensive care treatment in case of insufficient postoperative ICP control (Shim et al., 2023; Picetti et al., 2017; Shibahashi et al., 2023; Zhang et al., 2024). Although DC is intended to reduce ICP it does not necessarily eliminate the risk of persistent or recurrent postoperative intracranial hypertension. Previous studies have demonstrated that elevated ICP values may still occur after decompression and remain associated with neurological outcome (Picetti et al., 2017; Shibahashi et al., 2023; Zhang et al., 2024). Therefore, the role of continued ICP monitoring after DC remains clinically relevant, particularly in the early postoperative phase, where secondary brain injury may still evolve.

Previous studies have shown that postoperative ICP values exceeding defined baselines, such as 15 or 20 mmHg, are associated with unfavorable outcomes as measured by the Glasgow Coma Scale (GCS) or the modified Rankin Scale (mRS) (Sauvigny et al., 2018; Chen et al., 2022; Åkerlund et al., 2020a). Most studies on ICP monitoring and neurological outcome prediction have focused on isolated measurements such as mean ICP or peak ICP levels (Pan et al., 2020; Güiza et al., 2013; Mansour et al., 2023). Nevertheless, the cumulative postoperative burden of ICP, as quantified by metrics such as the area under the curve, has not been systematically investigated to date. Additionally, while preliminary efforts have explored the use of machine learning models based on ICP data to predict neurological outcomes, robust and clinically validated models remain scarce (Schweingruber et al., 2022).

The aim of the present study was to investigate the prognostic value of calculated intracranial pressure related area under curve (ICP-AUC) based on 72-h postoperative ICP monitoring following DC. Furthermore, we evaluated the predictive performance of machine learning models, including Random Forest and XGBoost classifiers, trained on patient-specific ICP data.

## 2 Materials and methods

This retrospective study included 17 patients who underwent DC due to severe brain injury resulting from the above-mentioned pathologies, followed by secondary cerebral edema. All patients were treated in a neurointensive care unit. The indication for DC was determined based on neurological status and radiologic signs. All patients received guideline-based neurocritical care aimed at controlling ICP prior to DC. Standard ICP-directed therapies, including sedation, osmotherapy and other supportive measures, were applied according to established clinical practice. Treatment strategies were not strictly standardized but were adapted to the individual clinical condition of each patient. Decompressive craniectomy was performed in patients with refractory intracranial hypertension despite these measures (Hawryluk et al., 2020; Hutchinson et al., 2016). Postoperatively, ICP was continuously monitored using a pressure sensor (Spiegelberg ICP monitor HDM 29.2). ICP values were recorded and automatically saved every 30 min. The duration of ICP monitoring varied between patients depending on clinical course and treatment decisions. In some cases, monitoring was discontinued earlier due to clinical improvement, while in others it was limited by severe deterioration. The monitoring period for prognostic analysis was set at 72 h. This time window was chosen to evaluate early cumulative ICP dynamics in relation to neurological outcomes, which were assessed using the mRS at discharge.

From the continuous ICP measurement, we calculated the ICP-AUC, maximum ICP values, and the percentage of time spent above 15 mmHg and 20 mmHg thresholds. The ICP-AUC was normalized by dividing the calculated AUC by the product of the maximum ICP value and the total monitoring time (72 h), resulting in a dimensionless measure of relative ICP burden.

Thresholds of 15 mmHg and 20 mmHg were selected based on the previous studies and clinical practice, as these values are considered relevant for prevention of secondary brain damage (Stein et al., 2023; Wijayatunga et al., 2022; Tan et al., 2015).

## 3 Outcome measurement

Neurological outcome was assessed using the mRS at hospital discharge, defined as the transition from acute inpatient care to rehabilitation or other subsequent care settings. The mRS is a widely used and validated measure of functional outcome across a range of neurological conditions (Banks and Marotta, 2007). The mRS scores were dichotomized into two groups: mRS <6 and mRS = 6, with an mRS score of 6 defined as death. The mRS scores were assessed by trained clinicians based on neurological status and patient records. Due the retrospective nature of the study, outcome data at discharge were not available for all patients, primarily due to incomplete clinical documentation. Due to the retrospective nature of the study, outcome data at discharge were not available for all patients, primarily due to incomplete clinical documentation.

## 4 Statistical analysis

To evaluate the prognostic value of early postoperative ICP dynamics, we analyzed the association between ICP measurements obtained during the first 72 h after DC and neurological outcomes (mRS at discharge). Correlation analyses (Spearman and Pearson) were performed for continuous variables, and dichotomized outcome groups (mRS <6 vs. mRS = 6) were compared to assess categorical associations. Additionally, a receiver operating characteristic (ROC) analysis was performed to evaluate the predictive value of early ICP measurements for unfavorable neurological outcomes. Statistical significance was set at a p-value of <0.05. All analysis were conducted using Python (Version 3.10) with packages *Pandas, Numpy, Statsmodels* and *Matplotlib*. All code used for the analysis and visualizations is available upon request.

## 5 Prognostic evaluation based on machine learning

A Random Forest Classifier and XGBoost model were trained using 72-h ICP measurements to predict poor neurological outcome or death, defined as mRS <6 vs. mRS = 6. Predictor variables included the normalized ICP-AUC, the maximum ICP value, and the percentage of time that ICP exceeded the baseline of 15 mmHg and 20 mmHg. Neurological outcomes were assessed at discharge. Model training and evaluation were performed using the *Scikit-learn* library in Python Feature contribution were visualized using Random Forest and XGBoost plots. To evaluate predictive performance, we used the following metrics: ICP-AUC, precision, recall and F1-score. Precision was defined as the proportion of correctly identified positive cases (e.g., mRS = 6) among all cases predicted as positive. It reflects how reliably the model predicts poor outcomes, aiming to minimize false positives. Recall, or sensitivity, was defined as the proportion of correctly identified positive cases among all actual positive cases. It evaluates how well the model detects all patients which poor outcomes, aiming to minimize false positives. The F1-score is defined as the mean of precision and recall. It provides a balanced single metric, especially useful when precision and recall are imbalanced or in datasets with class imbalance.

## 6 Results

### 6.1 Patient characteristics

A total of 17 patients were included in the study and all were included in the outcome-based analyses. Patients without complete outcome data were excluded from analyses. The median age at the time of surgery was 63 ± 11.4. of the patients, 12 (70.6%) were male and 5 (29.4%) were female. Additionally, 12 patients (70.6%) had anisocoric pupillary status preoperatively. 10 patients (58.8%) had cardiac comorbidities, and 6 patients (35.3%) had anticoagulation documented in their clinical records. Among, the patients, 16 (94.1%) had experienced a traumatic event. 1 patient (5.9%) developed a subdural hematoma (SDH) as a complication after the surgery. The types of traumatic brain injury in the cohort were as follows: 1 patient (5.9%) had a traumatic subarachnoid hemorrhage (SAH), 8 patients (47.1%) had traumatic SDH, 1 patient (5.9%) developed a postoperative traumatic SDH, 5 patients (29.4%) were diagnosed with both traumatic SAH and SDH, 1 patient (5.9%) had traumatic both SDH and intracerebral hemorrhage (ICH), and 1 patient (5.9%) had both traumatic SAH and epidural hematoma (EDH). The length of hospital stay was 15 ± 13.2 days. Patient characteristics are summarized in Table 1.

**Table 1:** Patient characteristics.

Median age at surgery ±SD	63 ± 11.4
Sex	
Female	5/17 (29.4%)
Male	12/17 (70.6%)
Preoperative anisocoric Pupillary status	
Yes	12/17 (70.6%)
No	5/17 (29.4%)
Cardiac comorbidities	
Yes	10/17 (58.8%)
No	7/17 (41.2%)
Anticoagulation documented in the medical history	
Yes	6/17 (35.3%)
No	11/17 (64.7%)
Initial diagnosis	
Traumatic SAH	1/17 (5.9%)
Traumatic SDH	8/17 (47.1%)
Postoperative SDH as complication	1/17 (5.9%)
Traumatic SAH and SAH	5/17 (29.4%)
Traumatic SDH and ICH	1/17 (5.9%)
Traumatic SAH and EDH	1/17 (5.9%)
Length of hospital stay in days ± SD	15 ± 13.2

The analysis of 72-h ICP measurements demonstrated a prognostic association with neurological outcome based on the established mRS. After dichotomizing our findings for analysis in mRS <6 and mRS = 6 the ICP-AUC showed a moderate positive correlation. Spearman correlation revealed r = 0.50 (*p* = 0.04), indicating a moderate association with poor outcome (mRS = 6) at discharge. In contrast, Pearson correlation demonstrated a weak and non-significant association (r = 0.27, *p* = 0.338). Among the evaluated parameters, ICP-AUC demonstrated the strongest association with neurological outcome among conventional ICP metrics, whereas mean ICP showed only a weak and non-significant correlation.

To further characterize the postoperative course of intracranial hypertension, we analyzed not only peak ICP values but also the duration of ICP elevation above clinically relevant thresholds. Patients with poor neurological outcome (mRS = 6) showed a higher cumulative ICP burden and spent a greater proportion of the 72-h monitoring period above the threshold of 20 mmHg. Notably, ICP values exceeding 20 mmHg for more than 30% of the observation period were significantly associated with mRS = 6 at discharge. These findings indicate that clinically relevant ICP elevation may persist despite DC and that both the magnitude and duration of ICP elevation are associated with outcome.

**Fig. 1:**
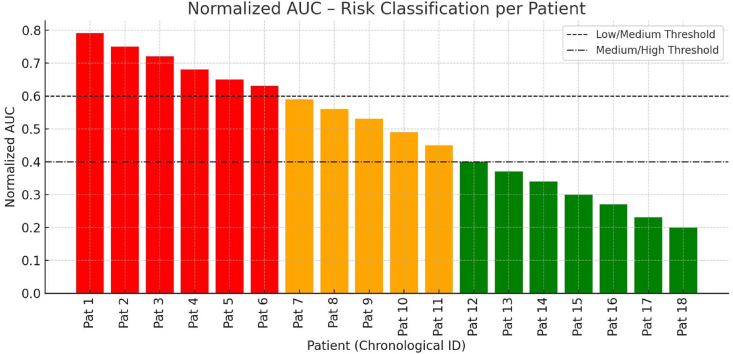
Patient-specific risk classification based on normalized AUC of ICP values within 72 h following DC. The bar chart illustrates the individual AUC values derived from continuous ICP monitoring over 72-h postoperative period. The AUC reflects the cumulative ICP burden, with thresholds of 15 mmHg (low/medium) and 20 mmHg (medium/high) used as reference values for analysis. Bar color indicated outcome-based classification according to the mRS at discharge: Red indicates poor outcome (mRS = 6, i.e., death), green indicates favorable outcome (mRS <6), orange represents an intermediate zone near the AUC threshold of 0.5, where outcome and ICP burden do not clearly correlate. Patients with an AUC ≥0.5 were generally associated with mRS = 6, indicating a high cumulative ICP burden and poor prognosis. Conversely, patients with an AUC <0.5 consistently demonstrated better outcomes (mRS <6).

**Fig. 2:**
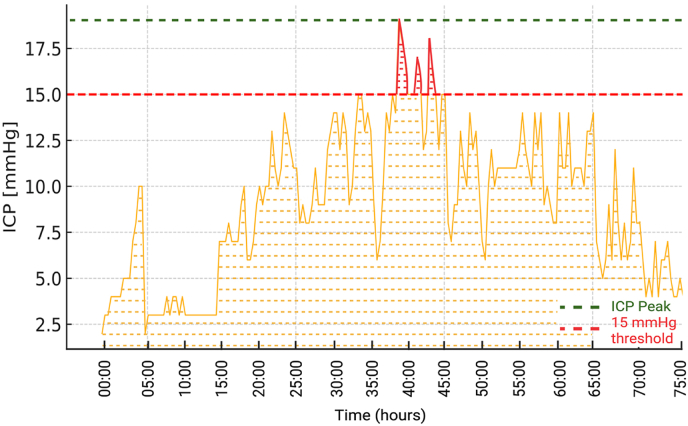
Example of ICP dynamics following DC. The ICP values were continuously monitored over a 72-h postoperative period. The dashed red line indicates the critical threshold of 15 mmHg. The highlighted area beneath the threshold represents the cumulative ICP burden, quantified as the area under the curve AUC. Increased cumulative ICP exposure, as visualized by the highlighted areas, was associated with poor neurological outcome, defined as mRS = 6 at discharge, in patients undergoing decompressive craniectomy.

The patient-specific assessment is presented in Fig. 1, which illustrates the normalized AUC for each patient and discusses the individual classification into risk categories in more detail. It is noteworthy that all patients in the red section of the diagram, except for one patient, exhibited an mRS = 6 at discharge, thereby indicating a poor neurological outcome. Furthermore, one patient who also had an mRS = 6, had only 50 h of ICP monitoring, which is comparatively brief when considered against the other patients in this study. A representative example of this analysis is shown in Fig. 2.

### 6.2 Discharge outcomes for threshold 20 mmHg

In view of the dichotomization between mRS <6 and mRS = 6, our findings indicate that an AUC ≥0.5 was associated with mRS = 6 in most cases. Notably, all patients with an AUC ≥0.6 showed an unfavorable neurological outcome (mRS = 6), whereas patients with an AUC <0.5 were significantly associated with mRS <6.

A *t*-test demonstrated that ICP thresholds of ≥20 mmHg were significantly associated with mRS = 6 at discharge (p = 0.03). In addition, a chi-square test confirmed the association between ICP levels ≥20 mmHg and mRS = 6. Our analysis further showed that when ICP values exceeded 20 mmHg for more than 30% of the 72-h monitoring period, this was significantly associated with mRS = 6 at discharge (*p* = 0.02).

### 6.3 Discharge outcome threshold 15 mmHg

Furthermore, analysis using a 15 mmHg ICP threshold confirmed a significant association with mRS = 6 (*p* = 0.047) at discharge.

### 6.4 Threshold comparison

Additionally, a chi-square test was performed to compare neurological outcomes (mRS = 6) at discharge between the thresholds of >20 mmHg and >15 mmHg. No statistically significant difference between the two thresholds was observed (*p* = 0.10). Furthermore, a Mann–Whitney *U* test was conducted using the same thresholds. This analysis demonstrated a significant association between ICP >20 mmHg and mRS = 6 (*p* = 0.017), as well as between ICP >15 mmHg and mRS = 6 (*p* = 0.031).

**Fig. 3:**
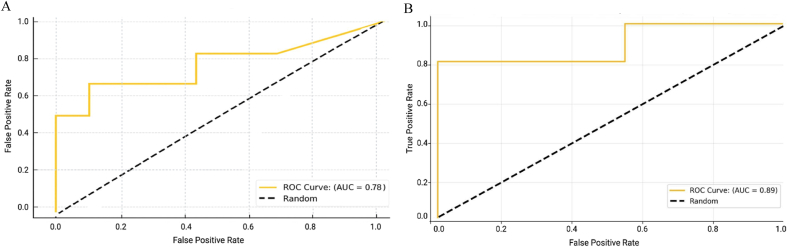
ROC curves assessing the predictive accuracy of normalized ICP-AUC for unfavorable neurological outcome (mRS = 6) at discharge following DC. **(A)** ROC curve for the threshold of 15 mmHg. The analysis is based on normalized ICP-AUC values derived from 72-h postoperative ICP monitoring across the entire patient cohort (n = 17). At this threshold, the AUC of the ROC-Analysis was 0.78, indicating moderate predictive accuracy in distinguishing between patients with mRS <6 and mRS = 6. **(B)** ROC curve for the threshold of 20 mmHg. At this threshold, the AUC of the ROC-Analysis increased to 0.89, demonstrating high predictive accuracy for identifying patients with poor outcome (mRS = 6) at discharge. In both ROC curves, the dashed diagonal line represents a random classifier (AUC = 0.5).

To evaluate the predictive accuracy of 72-h postoperative ICP measurements following DC for poor neurological outcome (defined as mRS = 6), a receiver operating characteristic (ROC) analysis was performed. ROC curves and the corresponding AUC values were calculated for the entire patient cohort (n = 17). Two clinically relevant thresholds, 15 mmHg and 20 mmHg, were selected for prognostic assessment. At the 15 mmHg threshold, the AUC was 0.78, indicating moderate discriminatory ability to differentiate between patients with mRS = 6 and those with mRS <6 at discharge. At the 20 mmHg threshold, the AUC increased to 0.89, demonstrating high predictive accuracy for poor neurological outcome (mRS = 6). Both ROC curves are presented in Fig. 3.

### 6.5 Machine learning model comparison

#### 6.5.1 Machine learning threshold for 20 mmHg

The Random Forest classification model, which used ICP-AUC, the maximum ICP and the percentage of ICP >20 mmHg for mRS = 6 demonstrated a high ICP-AUC of 0.89, a F1-Score of 0.80. The precision score for the Random Forest model was 1.0, which was defined that all patients with mRS = 6 were determined true at discharge. The Recall was 0.67, meaning that one third of the patients with mRS = 6 were not detected. Compared the XGBoost model, showed in all four metrics same values of 0.67. As well as ICP-AUC, Precision, Recall and F1-Score. Therefore, the XGBoost model demonstrated inferior performance in comparison to the Random Forest model, yet exhibited moderate predictive accuracy, albeit with a higher misclassification rate as well as mRS <6 and mRS = 6 outcome groups.

#### 6.5.2 Machine learning threshold for 15 mmHg

**Fig. 4:**
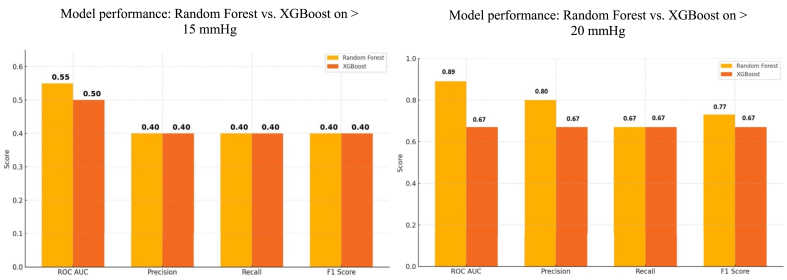
Comparison of the machine learning models Random Forest and XGBoost for the prediction of poor neurological outcome (mRS = 6) at discharge, based on normalized ICP-AUC values using two different thresholds. (A) Model evaluation at a threshold of 20 mmHg. The Random Forest classifier achieved an ICP-AUC of 0.89, precision of 1.00, recall of 0.67 and an F1-score of 0.80. In contrast, the XGBoost model yielded consistently lower values of 0.67 across all metrics, indicating reduced predictive performance. (B) At a threshold of 15 mmHg. The Random Forest model achieved an ICP-AUC of 0.55, while precision, recall and F1-score each reached only 0.40.

Additionally, a comparison of two machine learning models was also conducted to evaluate the predictive performance at an ICP threshold of 15 mmHg. The Random Forest model achieved an ICP-AUC of 0.55, indicating limited predictive validity. Additional performance metrics—including precision, recall, and F1-score—each reached a value of 0.40, reflecting low overall predictive accuracy. In comparison, the XGBoost model yielded an ICP-AUC of 0.50, consistent with random classification. Similarly, precision, recall, and F1-score were each 0.40, indicating no improvement over the Random Forest model and highlighting the limited utility of the 15 mmHg threshold for reliable outcome prediction in this setting. Fig. 4 summarizes the results of the Random Forest and XGBoost models.

## 7 Discussion

The aim of the present study was to evaluate the prognostic value of 72-h ICP monitoring in patients undergoing DC, with neurological outcomes assessed using mRS at discharge. For this purpose, the patient-specific dataset was dichotomized into two groups: mRS <6 and mRS = 6. Our findings suggest that patients with a normalized ICP-AUC >0.5 within the first 72 h postoperatively had a significantly higher risk of mRS = 6 at discharge. Additionally, the Random Forest classifier achieved a high accuracy in identifying patients with favorable outcome (mRS <6). While the machine learning model showed high predictive performance, this finding should be interpreted cautiously due to the limited sample size and potential overfitting. Furthermore, comparison of both thresholds used in this study (15 mmHg and 20 mmHg) did not show a significant difference in relation to mRS = 6 at discharge. Importantly, the study cohort represents a population with refractory intracranial hypertension, as all patients underwent DC following failure of standard ICP-directed therapies. However, ICP-directed therapy was not standardized across patients and was based on individual clinical decision-making, which may have influenced ICP dynamics. Compared to conventional ICP metrics, ICP-AUC demonstrated superior prognostic relevance, supporting the concept that cumulative measures of ICP may provide more clinically meaningful information than isolated values such as mean ICP.

Beyond its prognostic implications, our study also supports the continued clinical relevance of postoperative ICP monitoring after DC. Although DC is performed to reduce ICP, our findings suggest that elevated ICP values may persist during the early postoperative period and remain associated with poor neurological outcome. This observation is clinically important, as it challenges the assumption that decompressive surgery alone sufficiently eliminates the need for further ICP surveillance. Importantly, our AUC-based approach captures not only peak ICP values but also the duration and cumulative burden of intracranial hypertension, thereby providing a more comprehensive representation of postoperative ICP dynamics compared to conventional single-point measurements. In line with previous findings, time-dependent ICP metrics may offer greater prognostic value than conventional single-point measurements such as mean ICP (Åkerlund et al., 2020b; Güiza et al., 2015).

In their retrospective study, Sauvigny et al. investigated 102 patients with malignant middle cerebral artery (MCA) infarction and traumatic brain injury (TBI) who underwent decompressive craniectomy (DC), analyzing the association between postoperative ICP and neurological outcome. ICP was measured hourly over a period of 168 h. Their results showed a statistically significant difference between ICP thresholds of ≥15 mmHg and ≥20 mmHg in relation to unfavorable outcomes. Based on hourly ICP measurements, they identified critical ICP values ranging between 10 and 17 mmHg, which significantly impacted both 30-days mortality and functional outcome at the end of rehabilitation with a median follow-up of 129 days (Sauvigny et al., 2018). Despite a comparable methodology, the present study found no significant difference between ICP thresholds of 15 mmHg and 20 mmHg with to mRS = 6 assessed at discharge, based on ICP values measured during the first 72 h.

A comparable study by Hernández-Durán et al. involving 111 patients with malignant MCA infarction also performed ICP monitoring over a 72-h period. The primary aim of the study was to identify prognostic ICP thresholds for mortality. The authors found showed that a mean ICP greater than 10 mmHg was a strong predictor of mortality. Moreover, common clinical parameters such as age and initial GCS score were not significantly associated with survival (Hernández-Durán et al., 2021). Our findings support these results, as we demonstrated that a normalized ICP-AUC greater than 0.5 was significantly associated with poor neurological outcomes (mRS = 6). While Hernández-Durán et al. focused on mean ICP values, our study introduces AUC-based approach to capture the cumulative ICP burden dynamically over the same 72-h period. Both studies emphasize the critical importance of early postoperative ICP monitoring in predicting neurological outcomes following DC. Several studies investigated and highlighted importance of ICP monitoring after postoperative DC and the association with neurological outcomes based on GCS and mRS(Picetti et al., 2017; Chandankhede et al., 2023; Kim et al., 2014; Huang and Ou, 2016).

A key methodological difference from previous studies on postoperative ICP monitoring after DC lies in our use of a normalized ICP-AUC to quantify ICP dynamics over the initial 72-h period. While prior research often relied on mean, median and maximum ICP values to predict neurological outcome, our ICP-AUC based approach provides a cumulative, time-integrated, and dynamic measure of ICP burden (Picetti et al., 2017; Shahrom et al., 2024; Raj et al., 2022). This method captures both the magnitude and duration of ICP peaks, including the percentage of time above clinically relevant thresholds such as 20mmg and 15 mmHg. The dichotomization of patients based on normalized ICP-AUC threshold of 0.5 enabled us to define a practical and reproducible cutoff associated with mRS of = 6, which in our cohort reflected mortality. In contrast to previous studies that primarily employed regression-bases analyses, our study integrates as well as traditional statistical methods and machine learning models, allowing for improved predictive performance and clinical applicability in prediction of neurological outcome prediction based on 72-h ICP data.

The findings of the present study provide compelling evidence that ICP-AUC-based monitoring during the first 72 h following DC offers a valid and clinically meaningful prognostic marker for neurological outcomes at discharge. In our cohort, a normalized ICP-AUC greater than 0.5 was consistently associated with mRS = 6. This threshold may serve as a practical marker within dynamic ICP monitoring protocols to support clinical decision making.

Unlike previous studies that primarily focused on point specific ICP values such as mean or peak pressure our approach offers a cumulative prognostic assessment of ICP burden. Furthermore, our method enables risk stratification even in patients with unremarkable isolated ICP peaks by capturing the total duration and intensity of intracranial hypertension. As such, patients who show increasing ICP-AUC values over the defined threshold can be identified at an earlier stage, allowing for timely escalation of therapeutic interventions aimed at preventing latently developing secondary brain injury.

Prior studies, demonstrated that earlier and more targeted ICP monitoring and management correlates with improved neurological outcomes in neurointensive care (Stein et al., 2023; Baggiani et al., 2023; Addis et al., 2023; Le Roux, 2016). Conversely, patients with low cumulative ICP exposure (low ICP-AUC) may be spared from unnecessary or overly aggressive intensive unit treatment, which is also associated with adverse effects and reduced quality of life, particularly in the context of Post-Intensive Care Syndrome (PICS) (Dangayach et al., 2024; Hammer et al., 2021; LaBuzetta et al., 2019; Zhang et al., 2025). Furthermore, our study demonstrated that the machine learning, specifically a Random Forest classifier resulted in high predictive accuracy for identifying patients with mRS <6. While the number of patients was limited, the longitudinal nature of the data, with repeated ICP measurements over the 72-h period, resulted in a substantial number of data points per patient, which may support the use of machine learning approaches for integrating time-dependent features. These findings underscore the robustness of the ICP-AUC as a digital and objective indicator and support its potential for implementation in early-warning systems within neurocritical care management. Although only 17 patients had complete 72-h ICP measurements, we employed machine learning models (Random Forest and XGBoost) to explore early ICP burden dynamics in an exploratory manner. Statistical analysis using machine learning models in small patient cohorts is inherently challenging due to the risk of overfitting, especially when the number of observations (n) is smaller than the number of predictor variables (p). To mitigate this risk, we intentionally limited the number of predictors in our study to clinically meaningful features: the normalized ICP-AUC, the maximum ICP value, and the percentage of time ICP exceeded 20 mmHg.

For many machine learning algorithms, the condition n ≫ p is essential for achieving robust and generalizable performance. Despite this limitation, our study demonstrates that machine learning-based findings can serve as hypothesis-generating tools, and that data-driven risk stratification may still yield meaningful insights even with small cohorts when predictors are based on clinically relevant variable. Given the limited sample size, the predictive performance observed (ICP-AUC = 0.78 at 15 mmHg and ICP-AUC = 0.89 at 20 mmHg) should be viewed as exploratory findings, not as statistically confirmatory evidence.

The present study has several important limitations that should be considered when interpreting the results. First, the small sample size limits the statistical power of the analysis and reduces the generalizability of our findings. This is particularly relevant given the heterogeneity of underlying pathologies and clinical characteristics in patients undergoing DC. Established clinical prognostic factors, such as age, GCS, and pupillary status, were not systematically included in the analysis. This limits the ability to directly compare the relative strength of ICP-AUC with well-known predictors and should be addressed in future studies. Variability in ICP-directed treatment strategies may have influenced the observed ICP patterns and clinical outcomes, representing a potential source of bias. In addition, missing outcome data in a subset of patients represents a potential source of bias, especially in a small cohort. Although outcome data were available for the majority of patients, the absence of complete datasets may have influenced the observed associations. Furthermore, the application of machine learning models in small datasets carries an inherent risk of overfitting, particularly when the number of observations is limited relative to the number of predictor variables. Although we attempted to mitigate the risk by restricting the model to a small number of clinically meaningful predictors, the reported predictive performance should be interpreted with caution. In addition, the use of ICP measurements recorded at 30-min intervals may have limited the ability to capture short-term fluctuations or transient peaks in ICP. As a result, brief elevations in ICP may have been underestimated, which could influence the accuracy of cumulative metrics such as ICP-AUC. However, the longitudinal collection of data over a 72-h period resulted in a substantial number of measurements per patient, which may still reflect overall trends and the clinically relevant burden of intracranial hypertension. Furthermore, the aggregation of repeated measurements may contribute to increased robustness of derived features, particularly in the context of machine learning-based analyses. Withdrawal or limitation of care was not systematically assessed and may have influenced patient outcomes, representing a potential source of bias. In addition, ICP dynamics beyond the initial 72-h period were not assessed in this study. While this time frame captures the early postoperative phase, delayed ICP elevations may have been missed and should be considered in future studies. Furthermore, the duration of ICP monitoring varied between patients depending on their clinical course, which may have introduced additional variability in the analyzed date. In addition, neurological outcome was assessed at hospital discharge, which reflects early functional status rather than a standardized long-term outcome measure such as 3-month follow-up. This may limit the comparability of our findings with studies using established long-term outcome endpoints. Furthermore, the use of mRS instead of GOSe may limit direct comparability with studies focusing specifically on traumatic brain injury. Therefore, the findings of this study should be considered exploratory and hypothesis-generating rather than confirmatory. Larger, prospective and multicenter studies are required to validate the prognostic value of ICP-AUC and to establish the clinical utility of machine learning-based approaches in this setting.

## 8 Conclusion

In conclusion, the results of the present study suggest that 72-h postoperative ICP monitoring following DC, analyzed ICP-AUC metrics, provides a valid prognostic marker for neurological outcomes as measured by the mRS at discharge. Unlike previous studies, which primarily relied on single-time-point values or mean ICP, our findings demonstrate that a cumulative ICP-AUC-based approach allows for improved risk stratification. Moreover, the successful implementation of Random Forest classifier and XGBoost, showing high predictive performance, underscores the potential of machine learning tools for enhancing clinical decision-making in neurocritical care. Further multicentric studies with larger cohorts are warranted to validate the ICP-AUC-based monitoring approach and to establish the clinical utility of advanced machine learning models for accurate neurological outcome prediction.

## Consent to publish

Not applicable.

## Ethical approval and consent to participate

The study was approved by the Ethics Committee of the Medical University of Leipzig (approval 146/08-ek), which waived the requirement for written informed consent for this retrospective analysis of de-identified routine clinical data.

## Authors contributions

Data analysis: AEB, CG, VT; Manuscript drafting: AEB; Supervision: MV, Review Editing: JW, AEB, EG, TW; Design/Study: AEB.

## Data availability statement

The institutional clinical data generated and analyzed during this study are available from the corresponding author upon reasonable request.

## Funding

The present research received no funding

## Declaration of competing interest

The authors declare that they have no known competing financial interests or personal relationships that could have appeared to influence the work reported in this manuscript. No author has received honoraria, consulting fees, industry funding, or holds patents related to the content of this study. The research was conducted independently and without commercial or financial support.
